# Photo-Phytotherapeutic Gel Composed of *Copaifera reticulata,* Chlorophylls, and *k-*Carrageenan: A New Perspective for Topical Healing

**DOI:** 10.3390/pharmaceutics14122580

**Published:** 2022-11-24

**Authors:** Katieli da Silva Souza Campanholi, Ranulfo Combuca da Silva Junior, Renato Sonchini Gonçalves, Mariana Carla de Oliveira, Magali Soares dos Santos Pozza, Angela Tiago Leite, Leandro Herculano da Silva, Luis Carlos Malacarne, Marcos Luciano Bruschi, Leandro Dalcin Castilha, Tatiana Carlesso dos Santos, Wilker Caetano

**Affiliations:** 1 Chemistry Department, State University of Maringá, Maringá 87020-900, PR, Brazil; 2 Laboratory of Chemistry of Natural Products, Department of Chemistry, Center for Exact Sciences and Technology, Federal University of Maranhão, São Luís 65080-805, MA, Brazil; 3 Laboratory of Research and Development of Drug Delivery Systems, Department of Pharmacy, State University of Maringá, Maringá 87020-900, PR, Brazil; 4 Department of Animal Science, State University of Maringá, Maringá 87020-900, PR, Brazil; 5 Department of Physics, Federal Technological University of Paraná, Medianeira, 85884-000, PR, Brazil; 6 Physics Department, State University of Maringá, Maringá 87020-900, PR, Brazil

**Keywords:** photodynamic therapy, wound, healing, chlorophylls, carrageenan, copaiba oil-resin

## Abstract

Chronic wound healing represents an impactful financial burden on healthcare systems. In this context, the use of natural products as an alternative therapy reduces costs and maintains effectiveness. Phytotherapeutic gels applied in photodynamic therapy (PDT) have been developed to act as topical healing medicines and antibiotics. The bioactive system is composed of *Spirulina* sp. (source of chlorophylls) and *Copaifera reticulata* oil microdroplets, both incorporated into a polymeric blend constituted by *kappa-*carrageenan (*k-*car) and F127 copolymer, constituting a system in which all components are bioactive agents. The flow behavior and viscoelasticity of the formulations were investigated. The photodynamic activity was accessed from studies of the inactivation of *Staphylococcus aureus* bacteria, the main pathogen of hospital relevance. Furthermore, in vivo studies were conducted using eighteen rabbits with dermatitis (grade III and IV) in both paws. The gels showed significant antibiotic potential in vitro, eliminating up to 100% of *S. aureus* colonies in the presence or absence of light. The *k-*car reduced 41% of the viable cells; however, its benefits were enhanced by adding chlorophyll and copaiba oil. The animals treated with the phytotherapeutic medicine showed a reduction in lesion size, with healing and re-epithelialization verified in the histological analyses. The animals submitted to PDT displayed noticeable improvement, indicating this therapy’s viability for ulcerative and infected wounds. This behavior was not observed in the iodine control treatment, which worsened the animals’ condition. Therefore, gel formulations were a viable alternative for future pharmaceutical applications, aiming at topical healing.

## 1. Introduction

Wounds can be classified as a disruption in the tissue architecture, acting as a gateway for microorganisms that lead to local or systemic infections. Skin lesions affect people at any age; and can have different origins, such as trauma and ulcerative dermatitis. Several types of adjunctive treatment for acute wounds are available. However, cure rates are sometimes low, due to infections caused by resistant bacteria, causing secondary infections [1,2,3]. Therefore, developing research that deals with wound therapies is fundamental for emergent international hospital clinical protocols and guidelines based on updated scientific evidence [4,5,6].

A promising approach to reduce the burden of microorganisms is photodynamic therapy (PDT) [7,8,9,10], which is widely reported in the literature and available in hospitals in first-world countries. PDT is a minimally invasive and non-toxic antimicrobial strategy for killing infectious pathogens in an efficient, faster, and independent mode, in comparison with existing antibiotics [11]. This therapy is based on the excitation of a photosensitizer (PS) molecule by a specific wavelength [12]. The PS forms a long-lived triplet state that can react with molecular oxygen to generate reactive oxygen species (ROS), including singlet oxygen and hydroxyl radicals, leading, in situ, to cell death by necrotic and apoptotic modes [13,14]. As a result, significant advantages of PDT on antimicrobial chemotherapy are observed: it offers a short inactivation time to lead to cell death pathways; it does not lead to bacterial resistance after multi-course treatment; it does not cause dysbacteriosis; and it is applied to a wide range of target microorganisms, including Gram-positive and Gram-negative bacteria [7,9,15]. The widespread use of PDT in the treatment of skin tumors, actinic keratosis, acne, and rosacea is noteworthy and recognized by the Brazilian Society of Dermatological Surgery. In terms of contraindication, PDT is not indicated for the treatment of spots, as these are the skin lesions that have some chance of becoming melanomas, and the technique cannot be used on melanomas before surgical resection [16].

Another aggravating factor in wound therapy is related to the costs that result from treatments. For example, chronic wound healing in Brazil represents an impactful financial burden on the public health system, costing up to US$ 1772.40 per person (or treatment) [17]. In some countries, the financial costs of wound products are exclusively carried by patients, as there is no public health policy to support patients [18], which encourages researchers to search for effective and low-cost therapies. A therapeutic and inexpensive approach can be achieved with natural drugs and natural polysaccharides gels. In the last decade, several studies have been published concerning chlorophylls (Chls) as an antibiotic (PS compound) in PDT [9,19,20]. The advantage of natural Chls over synthetic PS is that the raw materials are readily available and inexpensive, and the extraction techniques do not require high labor costs [19]. In addition, chlorophylls absorb in the red region, the color of greatest skin penetration, which makes them attractive for topical use. In a recent publication, we showed the potential of Chls in PDT, which caused severe damage to the cell wall *Staphylococcus aureus* bacteria [9]. Here, the Chls from *Spirulina* sp. were from a microalgae that is widely used as a source of PS in PDT [21,22].

The benefits of Chls in PDT treatment can be harnessed through emulsions containing oil drops (emulgels), which provide preferential means of PS monomerizing. In this process, copaiba oil (CO) offers advantages in skin treatment, due to its cell regeneration properties [23,24,25,26,27], as widely recognized in popular culture. CO from the *Copaifera reticulata* genus is rich in sesquiterpenes and diterpene compounds [28]. Their composition reveals antimicrobial [29], larvicidal [30], repellent [31], antitumor skin disease healing [32,33], as well as anti-inflammatory properties [34]. The recognition of its potential led to the classification of CO as a medicinal plant of interest to the Brazilian Public Health System (SUS) and approval by the Food and Drug Administration (FDA) as a phytotherapeutic medicine [35,36]. Our research group has shown benefits in using copaiba oil from the *C. reticulata* genus, a natural product of northern Brazil [37,38].

Treating skin diseases requires using pharmaceutical forms that remain for a prolonged time at the site of action [8]. *Kappa-*carrageenan (*k-*car) is a natural sulfated polysaccharide with gelling properties. *K-*car has gained prominence in cosmetic and pharmaceutical applications, due to its healing, bactericidal, and thermoresponsive properties [39,40]. Additionally, F127 is a triblock micellar copolymer described as poly(ethylene oxide) (EO) and poly(propylene oxide) (PO) blocks, arranged as EO_99_PO_67_EO_99_ [41]. F127 unimers act as permeation promoters of *Copaifera reticulata* Ducke [38] and lead to Chls monomerization [9,42]. Therefore, this study identifies a polymer blend with therapeutic effect through the combination of *k-c*ar polysaccharide and F127 copolymer, with the aim of providing auxiliary healing and bactericidal activity to the incorporated drugs (phytotherapeutic gels; Phy-gel).

**Figure 1:**
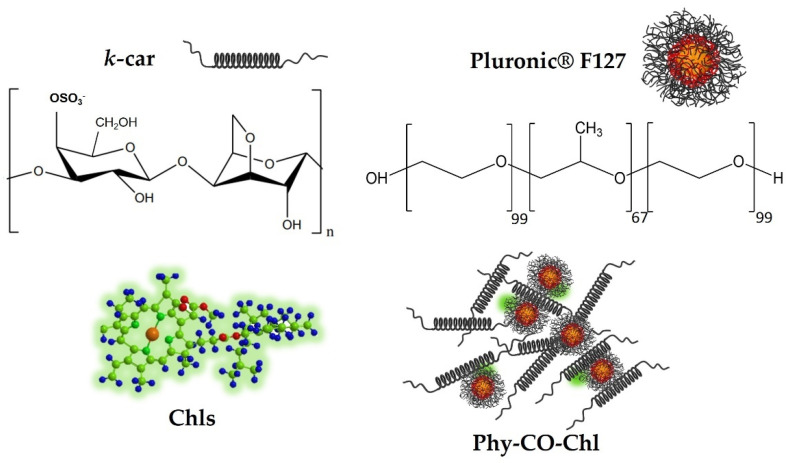
Obtaining the formulation composed of *k-*car, F127, chlorophylls, and CO, making up the Phy-CO-Chl emulgel.

Given the above-mentioned properties of Chls and CO, we present herein the design of a new platform-based *k-*car gel composed of *Copaifera reticulata* and Chls from *Spirulina* sp. as a phytotherapeutic (Figure 1) and safe alternative, with healing and antibiotic effects. Furthermore, rabbits with pododermatitis were used as a healing prototype to show the benefits of using PDT allied to Phy-CO-Chl (phytotherapeutic gel composed of copaiba oil and chlorophylls) as a new strategy for humans and for veterinary clinics.

## 2. Materials and Methods

The *Spirulina* sp. was purchased in local commerce and was used as acquired. Triethanolamine (TEA) was obtained from Synth (Diadema, São Paulo, SP, Brazil). Pluronic^®^ F127 (MW = 12,600 g.mol^−1^) and *k-*carrageenan were purchased from Sigma-Aldrich (St. Louis, MO, USA). Brain Heart Infusion Broth (BHI) and Mueller Hinton Agar (MHA) were purchased from Himedia (São Paulo, SP, Brazil) and KASVI^®^ (São José dos Pinhais, PR, Brazil), respectively. All experiments were carried out using ultra-pure water obtained from a Milli-Q system (Millipore, Merck, Darmstadt, Germany).

### 2.1. Copaifera reticulata Obtention

Copaiba oil-resin (*Copaifera reticulata*) was collected from geographic coordinates of −2.90344444 latitude and −49.47841667 longitude in the southwest of Pará state, Brazil. The management respected the ecosystem and sustainability of Amazonian biodiversity. The copaiba oil was extracted using a 1-inch hand drill for mechanical turning. The oil was extracted from holes drilled into the trunks of trees with a circumference greater than 1.20 m. The average collection was up to 1 L of oil resin from a selected tree, respecting the plant’s capacity. The hole was closed immediately after collection, remaining in this condition for 3 years, when a new collection in the same tree became viable [38].

The natural products were registered in the National Biodiversity Authorization and Information System (SISBIO nº 72922-1) and in the National System for Genetic Heritage Management (SISGEN nº A4F3A99).

### 2.2. Preparation of Gel Formulations

The gels were prepared using the previously reported methodology [43]. *K*-car and F127 copolymer (Table 1) were solubilized in purified water and homogenized using a mechanical stirrer for 20 min. Subsequently, *Spirulina* sp. (with or without CO) was added slowly into the polymeric pharmaceutical excipient. After complete homogenization, the formulation was kept refrigerated and protected from light. Formulations in the absence of CO and *Spirulina* sp. were also obtained, as shown in Table 1. The gel formulation, inserted in a glass bottle, was kept at rest for 24 h at 5 °C before further analysis.

### 2.3. Texture Profile Analysis

The texture assessments were conducted at 32 ± 2 °C (skin temperature) on the texture profile analysis (TPA). The emulgels’ mechanical properties were determined using TA-XTplus Texture Analyzer (Stable Micro Systems, Surrey, England), with a cylindrical probe (10 mm the diameter) moving at 2 mm.s^−1^. The probe performed two compressions, with 15 s of rest between them. Based on these measurements, charts displaying force values and displacement of the capital were obtained, which were used for determining the gel hardness, adhesiveness, cohesiveness, elasticity, and compressibility. The hardness (N) was obtained on the first positive peak maximum force. The elasticity (mm) was expressed by the quotient of the second/first peak. Cohesiveness was expressed by the quotient of the surface area under the first peak under the second positive peak. Adhesiveness corresponded to the surface area of the first negative. All the measurements were performed in at least six replicate samples for each formulation. The results are expressed as the mean ± standard deviation.

### 2.4. Rheological Analysis

The rheological properties of the dermatological emulgel were studied in a HAAKE MARS II rheometer (Thermo Fisher Scientific, Karlsruhe, Germany). The system was equipped with a parallel steel cone-plate geometry of 35 mm separated by a gap of 0.052 mm (cone code L09006 C60/1° Ti L). The gel was carefully applied to the lower plate, and the rheological profile information was collected after 1 min of stabilization. Continuous shear (flow) rheometry was investigated, with shear rates from 0 to 2000 s^−1^. The upward curve profiles were generated in a progressive increase in the shear rate up to 150 s, kept at the upper limit for 10 s, and progressively decreased throughout 150 s. Ostwald–de Waele and Herschel–Bulkley equations were used to adjust the replicated ascending flow curves, which obtained the values of consistency index (*K*), flow behavior (*n*), and yield value (σ_y_). The assessments were conducted at 32.0 ± 0.1 °C for at least three replicates.

The gel’s oscillatory performance evaluations were conducted in the interval of frequency sweep from 0.1 to 10.0 Hz, inside the linear viscoelastic region (LVR), in a condition of fixed stress. In this process, the parameters of dynamic viscosity (η’), storage modulus (G’), loss modulus (G”), and loss tangent (tan δ) were obtained using RheoWin 4.10.0000 (Haake^®^) software. The measurements were performed at 32.0 ± 0.1 °C. The elastic modulus (G’), the loss modulus (G”), the dynamic viscosity (η’), and the loss tangent (tan δ) were determined using RheoWin version 4.10.0000 (Haake^®^).

The stimuli-sensitive evaluation of systems was carried out by temperature ramp (from 5.0 to 60.0 °C), with a heating rate of 10 °C min^−1^. The analyses were conducted with a frequency of 1.0 Hz at LVR. All rheological analyses were performed using at least three replicates.

### 2.5. Photodynamic Evaluation

The photodynamic *Spirulina* sp. potential was performed by a qualitative method. The analyses were conducted using an ethanolic solution containing *Spirulina* sp. 4 mg/mL and 29 μmol/L of uric acid (UA). Uric acid is a chemical probe, and it degrades as PDT progresses. This solution was placed in a quartz cuvette and evaluated by collecting electronic absorption plots as a function of illumination time. The irradiation process for chlorophylls photoexcitation was carried out by a set of 6 red LED (5 mm model, λ_max_ = 632 nm, 12.5 mW), positioned evenly on the sides of the sample port of the spectrophotometer equipment UV-Vis (Varian, Cary 50 model), as previously reported [9]. The spectrophotometer works with modulated phase radiation, which allows the external red light to not influence the measurements.

### 2.6. Bacterial Photodynamic Inactivation: In Vitro Assays

#### 2.6.1. Microorganism Preparation

The *Staphylococcus aureus* (ATCC 25923) bacteria were activated in BHI broth. Before each experiment, the bacteria were replicated for two consecutive days and incubated at 37 °C for 24 h. The cell density was standardized in tubes containing a 0.9% sterile saline solution, and the McFarland scale was used for turbidity equivalence, corresponding to 10^8^ colony-forming units (CFU)/mL.

#### 2.6.2. Photodynamic Inactivation of *S. aureus* Performed by Chlorophylls Gel

**Figure 2:**
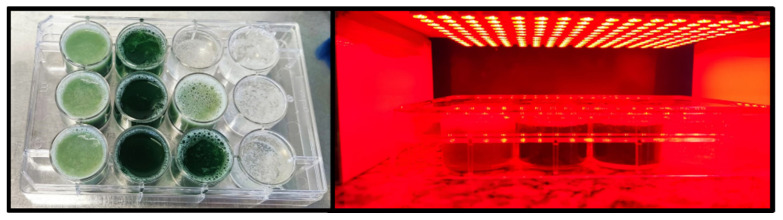
Illumination of Phy-Chl and Phy-CO-Chl gels.

First, as we previously reported, 1 mL of Mueller–Hinton broth and 3 g of phytotherapeutic emulgel were distributed in 12-well plates [44]. Next, the mixture was homogenized using a sterile tip. Then, 100 μL of the *S. aureus* suspension was added to each plate. The 12-well plates obtained, which had Phy-Chl and Phy-CO-Chl, were illuminated for 30 min with red LED (1.2 mw/cm^2^ with a light dose of 2.15 J/cm^2^, Figure 2). After illumination, 1 mL was collected from the well plates and added to Petri plates containing MH agar for subcultivation for 24 h at 37 °C. Finally, the controls were performed considering only bacteria, illuminated bacteria with red LED, Phy-Chl, and Phy-CO-Chl in the dark. After incubation, the total bacterial count, in CFU/mL, was determined [44,45].

### 2.7. Pododermatitis Treatment: In Vivo Assays in Rabbits

The study was previously submitted to the ethics committee on the use of animals, with approval under protocol 2268101221. All procedures performed in the study followed the international, national, and/or institutional guidelines for the care and use of animals. The animal experiment was carried out in the rabbit-farming sector of the Iguatemi Experimental Farm at the State University of Maringá.

#### 2.7.1. Animal Model

Rabbits (male and female between 2 and 3 years old, weighing approximately 3.5 kg and with paw lesions of grade III and IV) were used. Grade III lesions contain the presence of small scabs, sores, and moderate pain. Grade IV lesions contain infected wounds, with the presence of secretion and blood. The rabbits’ lesions were caused accidentally by the friction of the paws (without pads) in the breeding cages. Three animals per treatment, with lesions on both legs, were used (*n* = 6, considering the leg numbers). The initial dimensions for the Phy-gels treatment groups ranged from 1.20 to 3.1 cm. The group of control animals showed lesions between 0.54 and 1.98 cm. The treatment groups are shown in Table 2.

#### 2.7.2. Procedures

The animals were treated for 21 days with daily administrations of around 1 g of emulgel in each paw. The lesions were monitored every seven days and measured (diameter of lesions, Ø_day_), with pictures using ImageJ software. These results were expressed as a percentage of contraction, determined by $1-\left[\left(\emptyset_{21}*100\right)/\;\emptyset_{1}\right]$.

**Figure 3:**
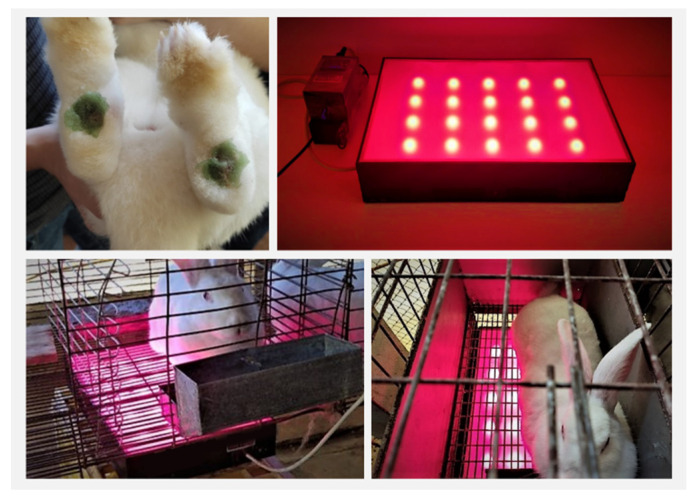
Gel administration and animal-illumination process.

The animals in the illuminated group were subjected to red light for 10 min. The lighting system was attached to the cages at a distance of 2.5 cm between the light and the animal. Red LED system (660 nm) was used as a flat platform (Figure 3), with a potential difference of 20.0 ± 0.5 V and the current *i =* 1.0 ± 0.1 A.

#### 2.7.3. Characteristics of the Lighting System

**Figure 4:**
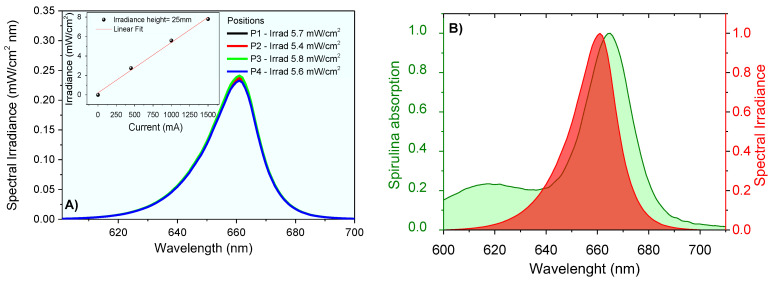
Spectral irradiance at 2.5 cm using different currents (**A**), and irradiance data linear correlations (insert); (**B**) spectral overlap between chlorophyll absorption and light source emission (normalized spectra).

The lighting system was designed for animal application. The irradiance was measured at the height of 2.5 cm for 4 different positions in the central region of the plate. The result shows an average irradiance $I=5.6\pm 0.2\;\mathrm{mW}/{\mathrm{cm}}^{2}$. At room temperature, the emission spectrum of the LED system was obtained using the UV-Visible Spectroradiometer System equipment of Gooch & Housego PLC, which was certified by the National Institute of Standards and Technology (NIST). The measurements provided the emission spectra from 250 nm to 800 nm wavelengths, and the integration method was used to obtain emission intensity values. The lighting system provided irradiance proportional to the current used (Figure 4) and the high spectral overlapped the absorption profile of the chlorophylls contained in *Spirulina* sp.

### 2.8. Histological Analysis

On the 21st day, one animal from each treatment was sent to the university abattoir after solid fasting (12 to 18 h). Slaughter occurred by electrical stunning and subsequent bleeding, following the current legislation for humane slaughter (*Resolution* n° 1000/2012 of the Federal Council of Veterinary Medicine, under Directive n° 47/2013 from *Ministério da Agricultura, Pecuária e Abastecimento*). During the slaughter procedures, the feet were separated and samples from the wound area were collected for histological analysis (both paws). Samples were fixed by immersion in 10% *w*/*v* formalin and processed by routine histological procedures to include samples in paraffin blocks [46]. Paraffin bocks were cut (3 µm) using an American optical 820 model microtome, and the slides were stained by hematoxylin-eosin (HE). The slides were analyzed via a Kasvi/Motic microscope with Motik Image proplus 2.0 software.

### 2.9. Statistical analysis

The averages were compared using the free software R, version 3.6.0, with the R studio interface, version 1.1.463 [47]. The statistical test was applied to compare the effect of *C. reticulata* and *Spirulina* sp. on the carrageenan gel properties in the oscillatory rheological behavior (at representative frequencies: 0.316, 1.000, 3.162, and 10.000 Hz), flow index, consistency index, hysteresis area, yield value, hardness, compressibility, adhesion, elasticity, and cohesiveness parameters. The significance level to reject the null hypothesis was 5% (*p* < 0.05).

## 3. Results and Discussion

Biopolymers for targeted drug delivery have been explored in many studies to develop the ideal dermatological biomedical platform. Carrageenans are sulfated biocompatible polysaccharides with prominent relevance in medicinal chemistry, pharmaceutical applications, and biotechnological research [48]. The *k-*car sulfate groups and F127 unimers offer polar and apolar environments for Chls monomerization. Furthermore, the combination of these polymers confers viscosity and adequate interfacial tension to avoid the coalescence processes of the CO droplets. The following analyses highlight the physical, rheological, microbiological, and curative aspects of phytotherapeutic gels (Phy-gels), with potential uses in human and veterinary medicine.

### 3.1. Physical and Chemical Aspects

**Figure 5:**
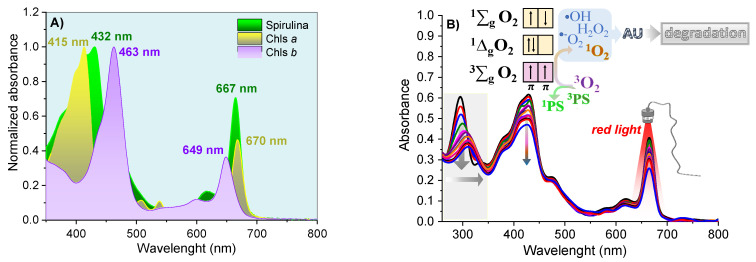
The normalized electronic absorption spectra for *Spirulina* sp. and purified Chls (**A**); and solution of *Spirulina* sp. 4 mg/mL and 29 μmol/L of UA in ethanol (**B**), under 70 min illumination with red light.

The Phy-gels were homogeneous, opaque and did not separate phases throughout the experiment. The *Spirulina* sp. had a high concentration of Chls *a* and *b* (and their derivatives compounds)*,* with an intense Q-band in the red region, between 645 and 695 nm (Figure 5A). The high concentration of chlorines showed photodynamic activity, a fact verified by the degradation of uric acid (UA, chemical probe) after activating the Chls compound. UA had a band at 294 nm and no bands in the visible region. The reduction of around 40% at 294 nm was related to the formation of reactive oxygen species formed after the light-activated Chls undergoes the process of inter-system conversion [8,9]. As shown in Figure 5B, the molecular oxygen in the ground state has a triplet state (^3^∑_g_). It has two low-lying singlet excited states, the ^1^∆_g_ (first excited state) and ^1^∑_g_ (second excited state), which differ in their electronic configuration of the π-antibonding orbitals. The transition from the ^1^∆_g_ state to the ^3^∑_g_ state is forbidden, leading to a long lifetime for the ^1^∆_g_ state [49]. On the other hand, the second excited state (^1^∑_g_) is short-lived, due to its permitted transition to the ^1^∆_g_ state. Most PDT compounds produce singlet oxygen (type II mechanism), which can oxidize the nearby biological species, such as lipids, amino acids, and DNA, due to the high reactivity of the unoccupied $\pi_{2p}^{*}$ from singlet oxygen. Type I mechanisms also occur (forming O_2_^•−^, •OH, H_2_O_2_), but with lower quantum yields. In the UA assays, all formed reactive oxygen species allowed its degradation, ensuring spirulina’s potential as a PS source in PDT. In addition to the decay of the UA band, the Chls absorptions were reduced by 24% due to photobleaching—chlorophyll degradation caused by radical species formed in PDT mechanisms [50,51].

**Figure 6:**
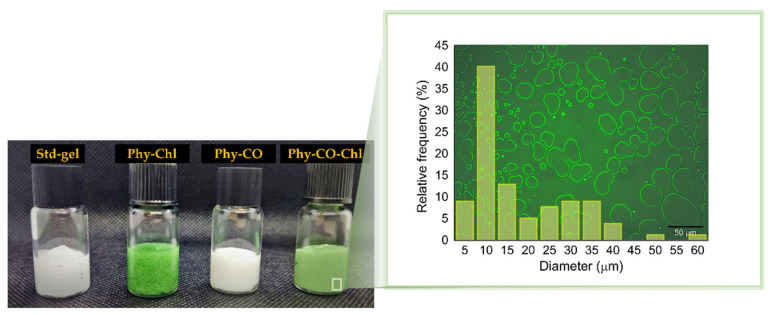
Photograph of the obtained k-car-based formulations. Std-gel corresponds to the polymer blend without drugs; Phy-Chl is the polymer base containing Chls, Phy-CO contains copaiba oil, and Phy-CO-Chl contains copaiba oil and Chls. The magnification represents the microstructure of the Phy-CO-Chl formulation.

After checking the in vitro photodynamic potential, the *Spirulina* sp. was combined with *C. reticulata* to obtain Phy-gels (Figure 6). Carrageenan is a known healing agent that acts as a structuring agent [52,53]. Pluronic^®^ F127 has been added in small amounts so that its unimers act as monomerization agents for hydrophobic PS, as previously shown [8,42]. Furthermore, F127 acts as a promoter of cutaneous permeation of C. reticulata, as we have recently shown [38].

The emulsions can be expressed by Gibbs free energy (ΔG), described by ΔG = ΔAγ-TΔS, being γ, the droplet interfacial tension, and ΔS, the entropy. The physical gel stability indicates that the cross-linked polymer (k-car) and the F127 polymeric surfactant reinforced the droplet copaiba oil interface, preventing coalescence in short periods. The smaller the droplet size, the better the thermodynamic stability the system acquires, according to Laplace’s law [54,55]. Therefore, the employment of a significant amount of energy to obtain small droplets is essential during manufacturing. The microstructure analysis of Phy-CO-Chl gel showed well-delimited interfaces and an accumulation of F127 on the drop’s surface (the lighter region in Figure 6). For the Phy-CO-Chl emulgel, 50% of the droplets were up to 10 μm in size [56]. We previously showed that Chls [8] and copaiba oil [38] have enhanced skin permeation with F127 micelles, reaching the dermis after 30 min of topical administration.

### 3.2. Mechanical and Rheological Properties

The rheological and textural studies can estimate the suitability of the gel platforms under industrial manufacture and also in environmental and physiological body conditions [38]. The data from the textural mechanical analysis are displayed in Table 3.

The hardness values reflect the force required to produce deformation in the semi-solid system. The gels exhibited a reduction in this parameter with the CO and *Spirulina* sp. addition (*p* < 0.05), due to the influence on the polymeric chain interactions [10]. The adhesiveness property may indicate the potential interaction of formulation with the skin after administration. The Phy-gels showed variations without statistical significance (*p* > 0.05) for this parameter. However, all systems proved to be an adhesive interface, an essential feature for wound closure at joints and other moving parts. The compressibility showed significant reduction (*p* < 0.05), with Chl components and variations without statistical relevance after CO addition (*p* > 0.05). Cohesiveness and elasticity are related to the ability of the system to restructure after successive deformations. For these parameters, no significant changes were verified with the variation of the formulation’s composition (*p* > 0.05). Furthermore, all gel formulations showed property values that were similar to those of commercial products consolidated in the market, as previously reported (Table 4) in other studies about k-car gels [57,58].

**Figure 7:**
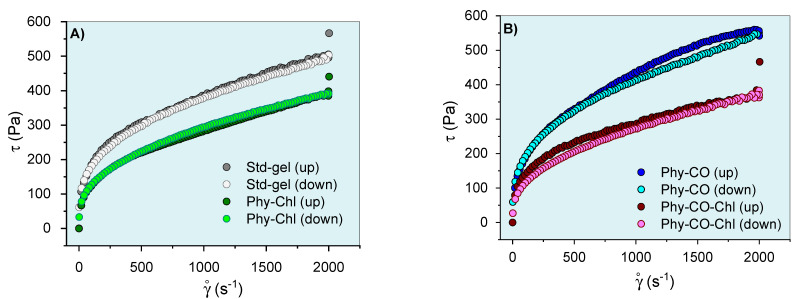
Phy-gels continuous flow rheograms at 32.0 °C (**A**,**B**). Standard deviations were omitted for clarity; however, the coefficient of variation of the replicate analyzes was <10% in all cases.

After knowing the gel’s mechanical properties, the flow rheograms were investigated. Rheological studies can characterize the flow behavior of a formulation and allow the understanding of the structuring and interactions between the components of the gel. The continuous flow rheograms are shown in Figure 7.

The flow rheograms (Figure 7A, B) showed the non-linear relationship between the applied shear stress (τ) and the shear rate (velocity gradient in reciprocal time units—ẏ). In this process, the viscosity coefficient represents the proportionality relationship between the τ and the ẏ. Its values (angular coefficient) were higher at lower oscillatory frequencies and became lower as the shear rate progressed. The viscosity reduction shown in Figure 7 resulted in an *n* exponent (flow behavior index) <1, and the gel can be classified as a non-Newtonian and pseudoplastic system [8,61,62]. This conduct can be explained by the high entanglement and friction of the *k-*car and F127 chains in the resting state, which allow higher apparent viscosity. However, the application of tension leads to the alignment of the polymer chains, which reduces viscosity. Its behaviors are typical of semi-solid systems [8,63]. The incorporation of *Spirulina* sp. (Phy-Chl and Phy-CO-Chl, as shown in Figure 7B) reduced the overall viscosity of the system; data concordant with structural change showed in the textural parameters (Table 3), which may be linked to reduced interaction between the polymer and polysaccharide chains. The CO effect, which remains restricted to the micellar core as a droplet, increased the global viscosity of the Phy-CO gel, an effect already verified for *C. reticulata-*loaded in semi-solid systems [38]. The application of shear stress forces to the Phy-CO gel allows the unraveling of chains and reduces the droplet size to the critical rheological point, which reduces overall viscosity [38,64,65,66]. Most of the flow rheograms showed no hysteresis area, except the Phy-CO gel, which presented little thixotropy, due to the oil droplets. The Phy-CO-Chl gel showed rheopexy indicative (negative hysteresis area), due to the finite time nature of the analysis.

The Ostwald–de Waele and Casson equations modeled the upward curves for statistical analyses. A flow behavior index (*n*) below unity was obtained (*n* from 0.32 to 0.40 values for all systems, as shown in Table 5) and showed an increase with the drug incorporation (*p* < 0.05). Furthermore, the consistency index (*K*) and the yield value (σ_y_) decreased up to 50% with the Chls addition, and up to 19% with the CO incorporation (*p* < 0.05). The σ_y_ values indicated the need for a prior force to start the lamellar flow. The data obtained for this parameter were typical of semi-solid systems and indicated an adequate capacity of the gel to remain at the site of action, in addition to the preventive capacity to destroy the structure of the emulsion (coalescence of the droplets) [67]. Campanholi et al. (2022) [38] showed complex rheograms from *Copaifera reticulata* Ducke in drug-delivery systems for topical use (blends of F127 and carbopol C934P). The complex nature and difficulty of upward curve modeling were attributed to the complexity of the gel structure, which suffered constant effects of the droplet redistribution during shear.

**Figure 8:**
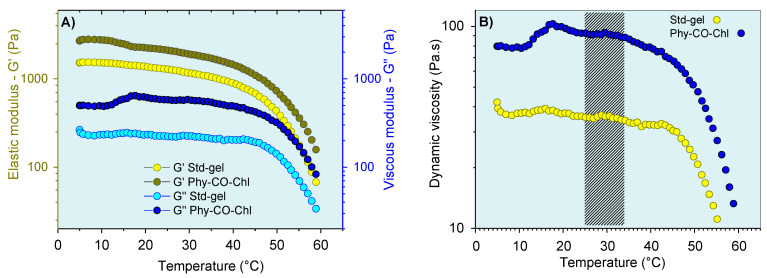
Viscoelastic properties of the gel formulations as a function of temperature: (**A**) storage modulus (G’) and (**B**) dynamic viscosity. The marking in (**B**) corresponds to the variation between temperatures of 25 °C (room temperature) and 32 °C (skin temperature). Standard deviations were omitted for clarity; however, the coefficient of variation of the replicate analyzes was <10% in all cases.

The thermoresponsive nature of the *k*-car was also subjected to oscillatory analysis (Figure 8). The CO and Chls incorporation into Std-gel led to an increase of the G’ (elastic) and G” (viscous) moduli at all temperatures, a behavior already reported for carrageenan [68]. In addition, the systems showed viscoelastic behavior (G’ > G”), with a pronounced viscosity reduction above 45 °C. Therefore, the thermo-responsive properties of these compositions can be advantageous in the gel-preparation step, which can be more easily obtained at 45 °C. However, with the administration process, the temperature variation (7 °C) does not substantially change the behavior of the formulation, as shown in Figure 8B.

**Figure 9:**
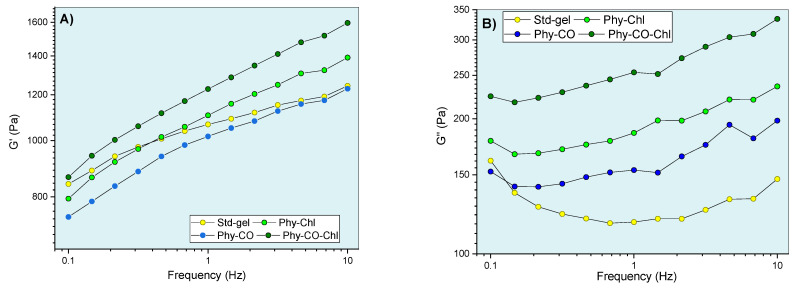

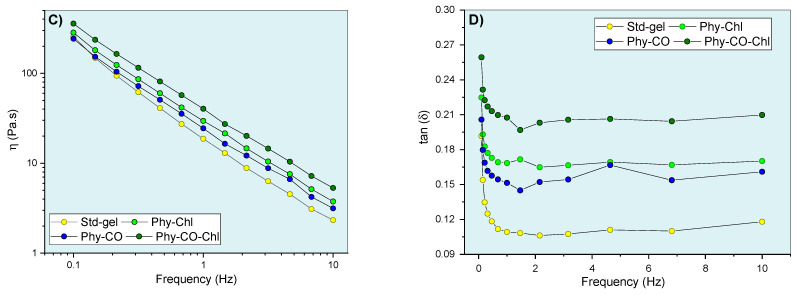
Gel oscillatory properties at 32.0 °C, being (**A**,**B**) G’ (elastic) and G” (viscous) moduli, (**C**) dynamic viscosity, and (**D**) viscoelasticity properties as a function of oscillatory frequency.

Regarding oscillatory rheometry, an analysis within the linear viscoelastic region (fixed stress values ranging from 2.5 to 8 Pa, depending on the formulation) was employed at skin temperature (32 °C) (Figure 9). This region allows infinitesimal deformation, and the biopolymer chains are kept close to equilibrium. Thus, the oscillatory responses showed molecular-level interaction information. The viscoelastic properties were verified by obtaining the G’ and G” moduli. Their relationship, expressed as G”/G”, showed viscoelastic behavior when the values were less than one. In this condition (G”/G’ < 1 ), there was a predominance of elastic interactions and of reversible nature [69].

The viscoelastic behavior was dependent on composition and frequency. The CO and Chls incorporation (Phy-CO and Phy-Chl) did not significantly affect the G’ modulus (*p* > 0.05). On the other hand, the Phy-CO-Chl obtention increased the G’ modulus in a pronounced way, especially at the highest frequencies (*p* < 0.05). The G” modulus showed slight increases in the following order: Std-gel, Phy-CO, Phy-Chl, and Phy-CO-Chl. Nevertheless, the increases were not statistically significant (*p* > 0.05). The viscous moduli increase was of greater magnitude than the elastic moduli increase, thus enhancing the plastic characteristic of the formulations. An elastic deformation nature may be related to drug/copolymer interaction, while plastic deformations may be related to changes in the helical conformation of the *k-*car biopolymer. The dynamic viscosity was reduced with small movements (low oscillatory frequencies). However, above 6 Hz, the variation was less pronounced. The gel composition did not cause significant changes in the dynamic viscosity (*p* > 0.05).

The tangent loss, expressed as tan δ (G”/G’), decreased with the oscillatory frequency increase (*p* < 0.05) for all systems. The enhanced viscoelasticity followed the complexity of the matrix composition, where Phy-CO-Chl showed tan δ of 0.259, a nearly two-fold increase compared with that of Std-gel. All formulations had tan δ values lower than one (Figure 9D), and were classified as viscoelastic systems. This is an essential property when considering drug-delivery systems, as it suggests an appropriate retention of these formulations at the application site [63,69,70,71,72,73,74].

### 3.3. Photodynamic Inactivation of Staphylococcus Aureus Bacterial

**Figure 10:**
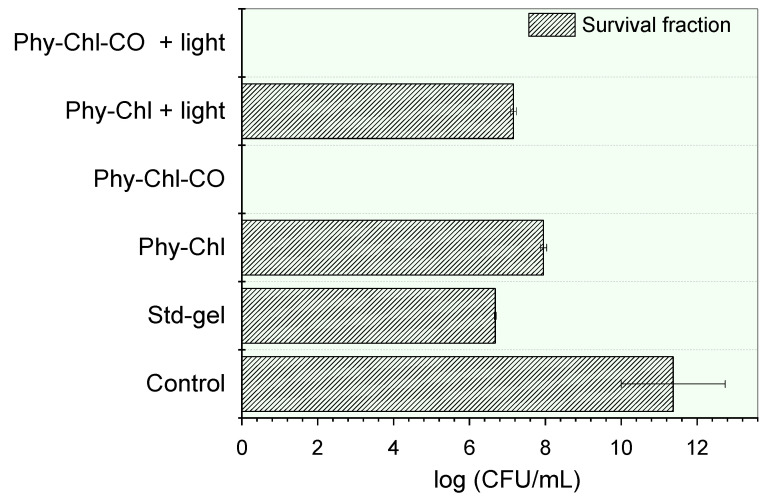
Inactivation of S. aureus by a set of phytotherapeutic gels applied to PDT. The absence of bars indicates the complete elimination of the microbial load.

Wound healing involves three major phases: inflammatory reaction, cell proliferation, and remodeling. First, the inflammatory process occurs with vasoconstriction, hemostasis, and inflammatory mediator release. Then, the granulation tissue is triggered, with fibroblast proliferation and angiogenesis effects. At this point, opportunistic pathogens, such as *Staphylococcus*, infect small dermal lesions and invade subcutaneous tissue [46,75,76]. Therefore, inactivation studies of these pathogens are fundamental to evaluate the healing and antibiotic potential of the drug. The antibacterial activity of Phy-gels against *S. aureus* is presented in Figure 10.

Bacterial cell incubation with gels led to statistically significant count reductions of the colony-forming units (CFU) (*p* < 0.05). The Std-gel showed bacterial potential, with a countdown of 41%, a similar result to that previously reported [77]. The Chls incorporation further reduced the count of viable cells count (*p* < 0.05). The Phy-Chl activity without light showed a 30% CFU reduction, and this value increased to 37% after light. The CO addition into the matrix gel increased the potential of the formulation, allowing the total elimination of all viable cells in the light and in the dark. This pronounced increase was probably related to the bactericidal behavior of the CO and its influence on the Chls additional monomerization capacity. The interface-defined droplets can solubilize hydrophobic PS in their microdomains, a known behavior for emulgels with hydrophobic compounds [67]. The higher rate of monomers increases the number of molecules able to undergo intersystem conversion, so that singlet oxygen (the PDT protagonist) is formed.

Although in vitro treatment suggested efficiency in the absence of light for the Phy-CO-Chl gel, photodynamic treatment is essential, because Chls inhibit the resistance effect on pathogens by generating singlet oxygen. At the same time, CO acts as a wound-healing agent [9,37,38,44,78]. We have previously shown severe damage that low concentrations of Chls can cause to the bacteria wall, making its efficiency in PDT unquestionable [9]. Moreover, the literature reports that red wavelength LED significantly enhanced the skin graft score, which increased the transforming growth factor beta (TGF-β) protein expression and the density of collagen fibers [79]. Therefore, at the same time as the drugs act, red LED enhances the dermo–epidermal junction and modulates the expression proteins related to tissue repair [79].

The combined mechanical, rheological, and photo-antimicrobial benefits encouraged in vivo studies on animals with wound lesions.

### 3.4. Photodynamic Treatment: In Vivo Assays in Rabbits

The benefits of carrageenan and copaiba oil in wound healing are already known [48,80,81,82], but not widely reported with the addition of Chls.

The lesion shrinkage is shown in Table 6. The values, measured at 7 day intervals, were expressed in percentages, considering the first and last day of the treatment. The mean shrinkage value of the wounds treated with the formulations varied between 59.5% and 100%.

The lesion contraction effect between the groups treated with Phy-gels was statistically similar (*p* > 0.05), but different from that of the iodine treatment (*p* < 0.05). For lesions submitted to the control treatment, 80% showed an increase in lesion severity, and only 20% had their lesions reduced by up to 37%. Although the phytotherapeutic treatments (light and dark) were equivalent, the PDT reduced the pathogen load without generating bacterial resistance effects, which inhibited the systemic level of infection or the development of chronic wounds [7,9,10].

**Figure 11:**
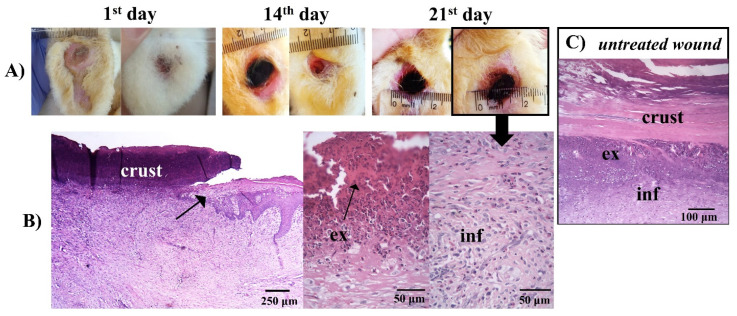
(**A**) Macroscopical aspects of the plantar face of feet in rabbit breeders during control treatment. (**B**) Histological appearance on the lesion areas in the last day of treatment with iodine. The arrow in the photo indicates the border of the wound. The wound area surface was characterized by debris and exudate (ex), and a concentration of polymorphonuclear and inflammatory cells (inf) in the dermis. (**C**) Photomicrographs of histological aspects of untreated wounds. (**B**,**C**) Paraffin section, HE.

The worsening of the clinical condition of the iodine-treated animals can be seen in the macroscopic images of the rabbits’ feet and their respective histologies (Figure 11).

The histology in Figure 11 showed a similarity between the histology of the iodine-treated (Figure 11B) and the untreated animals (Figure 11C). Injuries tissue (Figure 11A), vasodilatation, and migration of inflammatory cells to the lesion region, due to the attraction of chemopathic molecules, can be verified (Figure 11B and Figure 11C). Furthermore, there was an increase in space between the connective tissue cell fibers, due to the increased interstitial fluid resulting from edema, as described by other authors [80,83].

The treatment with Phy-gel was more effective than that of the control. In all cases treated with Phy-gel, the healing progression was concordant with the initial degree of wound healing.

**Figure 12:**
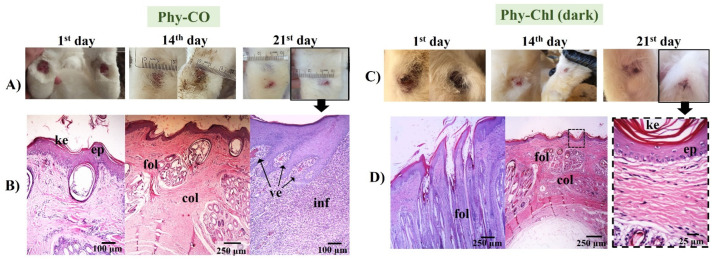
(**A**,**C**) Macroscopical aspects of the plantar face of feet in rabbits during Phy-CO and Phy-Chl (dark, without PDT use) treatment. (**B**,**D**) Histology of the last day of each treatment. The arrow from the photo of the feet indicates that the paw was analyzed histologically. The images show the keratin layer (ke), the stratified keratinized epithelium (ep), collagen presence (col), hair follicles (fol), blood vessels (ve), and signs of inflammation (inf). (**B**–**D**) Paraffin section, HE.

The Phy-CO formulation showed almost complete healing by the 21st day. However, the epithelium was still thick, with a thin keratin layer. The dermis was in a phase of organization, while the hair follicles were organized, with signs of hair emergence [46]. However, as seen in the Figure 12, healing was not complete, and inflammatory cells were still found in specific segments, with blood containing leukocytes accumulating (Figure 12). The benefits of CO as a healing and anti-inflammatory agent have already been reported in oral or topical administration [25].

The treatment with Phy-Chl (dark) favored healing, which was maintained on 21^st^ day with only slight edema (Figure 12). The treated lesion tissue showed intact epithelium, with reorganized dermis and hair follicles. Moreover, fur began to develop and emerge. There are few studies on the healing capacity of Chls [84,85], with a few occurring in the 1950s. The results presented here support the potential for combining Chls and k-car. The Phy-Chl treatment showed more promise than Phy-CO for the tested treatment interval.

**Figure 13:**
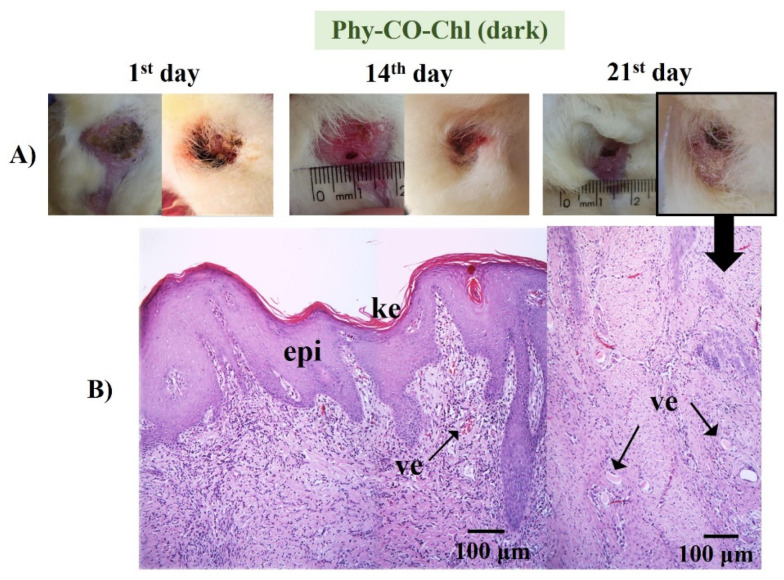
(**A**) Macroscopical aspects of the plantar face of feet in rabbits during the Phy-CO-Chl treatment, without PDT use. The arrow in the photo of the feet indicates that the paw was analyzed histologically. (**B**) Histological aspect of skin in the last day of treatment (paraffin section, HE). The images show the keratin layer (ke) and the presence of tissue vascularization (ve).

Phy-CO-Chl (Figure 13) treatment showed edema at the end of treatment, with the presence of inflammatory cells in the dermis region, broken follicles, and the presence of blood accumulation due to the effect of vasodilation. The treatment variations (all Phy-gels in dark conditions) were linked to deep lesions and animal management, as the animals were kept in cages during treatment, which made healing difficult due to the constant friction of the feet in the cages. Overall, the treatments showed benefits of the Phy-gels (without the use of PDT) for healing, even if the benefits were incomplete (an increase in the frequency of dosage or treatment time could lead to complete healing). On the other hand, the treatment with iodine worsened the condition of all animals. The results obtained in the dark were similar to those reported by Plefh et al. (2021) [46], who evaluated 21 day treatments with a fluid gel containing 2% of clove powder (*Syzigium aromaticum*), as antimicrobial, antioxidant, and anti-inflammatory properties on wounds (pododermatitis) of rabbits.

**Figure 14:**
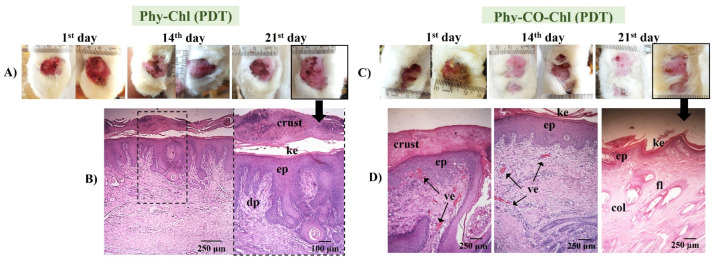
(**A**,**C**) Macroscopical aspects of the plantar face of feet in rabbits during Phy-Chl and Phy-CO-Chl (light, with PDT use) treatment. (**B**,**D**) Histology in the last day of treatment, evidencing the dermic papillae (dp), the keratin layer (ke), the stratified keratinized epithelium (ep), wound crust, the presence of vascularization of the tissue (ve), the presence of collagen (col), and hair follicles (fol).

PDT use in pododermatitis cases aims to reduce the pathogen load. Furthermore, the short lifetime of singlet oxygen does not allow antimicrobial resistance [7,9,86]. The activation of the photochemical and photophysical mechanisms was possible due to the overlap of the chlorophyll absorption spectrum (Q-band at 670 nm) with the emission from the red source (660 nm). The evolution of the lesions submitted to PDT is shown in Figure 14.

Phy-Chl+PDT showed superior healing signs, with the regeneration of the dermal papillae and the formation of a thick keratin layer. Furthermore, the treatment favored the regeneration of the epithelium, which was intact on the last day of treatment. Similarly, the Phy-CO-Chl+PDT exhibited a process of reepithelization in addition to the formation of fur. Furthermore, the presence of a crust was verified in the lesion region (according to the 21st day photograph, as shown in Figure 14), indicating that the healing process was still in progress [48,80]. The improvement in the results is linked to the tissue-recovery capacity stimulated by the red light, which is associated with the reduction of the pathogen loads that cause infection (Figure 10). In addition, the benefits of using red light in the reepithelialization process are known, with light being a treatment mechanism (photobiomodulation to improve immune system function) [87,88,89].

## 4. Conclusions

The chlorophyll from *Spirulina* sp. showed photodynamic activity by degrading uric acid in a homogeneous medium (ethanol). The gel composed of *Spirulina* sp. and *Copaifera reticulata* exhibited evident bactericidal capacity, as it caused the death of 100% of *S. aureus*, whether in the presence or absence of photodynamic therapy. Moreover, the phytotherapeutic gels were viscoelastic, adhesive, cohesive, and pseudoplastic, being essential for drug-delivery topical systems. In vivo studies proved the potential for phytotherapeutic gels, showing a lesion contraction effect with evidence of healing and reepithelialization in histological analyses. The use of iodine (control) worsened the clinical picture of the animals, with pronounced enlargement of lesions and signs of severe infections. Therefore, the gel formulation associated with photodynamic therapy showed satisfactory potential, representing a viable alternative for pharmaceutical applications aimed at topical healing.

## Figures and Tables

**Table 1:** Composition of the topical formulations.

Composition	Formulations (%, *w*/*w*)			
	Gel-Std	Phy-Chl	Phy-CO	Phy-Chl-CO
Carrageenan	2.0	2.0	2.0	2.0
F127	4.0	4.0	4.0	4.0
*Spirulina* sp.	0	0.5	0	0.5
*Copaifera reticulata* (CO)	0	0	4.0	4.0
Purified water	94	93.5	90	89.5

**Table 2:** Rabbits’ treatment groups.

	Treatment Group
Dark	Phy-Chl
	Phy-CO
	Phy-CO-Chl
	Iodine 10 %, m/V (control)
Light	Phy-Chl
	Phy-CO-Chl

**Table 3:** Textural mechanical characteristics (hardness, adhesiveness, elasticity, cohesiveness, and compressibility) of gel formulations (mean ± standard deviation).

Parameter	Std-Gel	Phy-Chl	Phy-CO	Phy-CO-Chl
Hardness (N)	0.238 ± 0.009	0.149 ± 0.005	0.184 ± 0.004	0.171 ± 0.025
Adhesiveness (N.mm)	1.033 ± 0.127	0.681 ± 0.048	0.919 ± 0.032	0.881 ± 0.047
Elasticity (mm)	1.001 ± 0.003	1.002 ± 0.005	0.999 ± 0.003	1.001 ± 0.005
Cohesiveness (dimensionless)	0.647 ± 0.063	0.742 ± 0.023	0.680 ± 0.011	0.695 ± 0.034
Compressibility (N.mm)	2.404 ± 0.188	1.472 ± 0.116	2.050 ± 0.075	1.863 ± 0.238

**Table 4:** The mechanical properties of commercial gel formulations.

Parameter	Values	Commercial Gel	Reference
Hardness (N)	0.41 ± 0.02	Emo^®^	[59]
	0.15 ± 0.01	Opokan^®^	[59]
Adhesiveness (N.mm)	0.57 ± 0.06	Opokan^®^	[59]
Elasticity (mm)	0.96 ± 0.06	Kenacort-A Orabase^®^	[60]
Cohesiveness (dimensionless)	0.83 ± 0.04	Emo^®^	[59]
Compressibility (N.mm)	1.92 ± 0.13	Kenacort-A Orabase^®^	[60]

**Table 5:** Consistency index (*K*), flow behavior (*n*), and yield value (σ_y_) obtained with upward flow curve modeled by Ostwald–de Waele and Casson equations.

Rheological Parameters	*K* (Pa.s)^n^	*n* (Dimensionless)	σ_y_ (Pa)
Std-gel	37.51 ± 2.70	0.32 ± 0.02	127.65 ± 10.02
Phy-Chl	18.90 ± 2.27	0.40 ± 0.00	82.80 ± 8.97
Phy-CO	30.29 ± 2.92	0.39 ± 0.01	130.67 ± 17.14
Phy-CO-Chl	22.72 ± 6.88	0.34 ± 0.05	106.66 ± 33.41

**Table 6:** Average lesions (percentages) contraction between the 1st and 21st day, considering six legs for each treatment.

Treatment	% Wound Contraction	
Phy-Chl	86.0 ^a^	Dark
Phy-CO	100.0 ^a^	
Phy-Chl-CO	59.5 ^a^	
Phy-Chl	87.0 ^a^	Light
Phy-Chl-CO	84.0 ^a^	
Iodine control	−78.3 ^ab^	

Equal letters are equivalent to *p* > 0.05, and different letters to *p* < 0.05.

## Data Availability

Not applicable.
